# Frederic Lewy: how the two World Wars changed his life, work, and name

**DOI:** 10.1055/s-0044-1779692

**Published:** 2024-03-11

**Authors:** Patrick Emanuell Mesquita Sousa-Santos, Pedro Machry Pozzobon, Igor de Lima e Teixeira

**Affiliations:** 1 Universidade Estadual Paulista “Júlio de Mesquita Filho”, Botucatu SP, Brazil.

**Keywords:** Frederic Lewy, Inclusion Bodies, Lewy Bodies, Frederic Lewy, Corpos de Inclusão, Corpos de Lewy

## Abstract

In 1912, Friedrich Lewy described the inclusion bodies present in Parkinson disease and in Lewy body dementia. Throughout his life, Lewy fought in two wars – on opposite sides. He was born in Berlin in a Jewish family, and served in the German Army in World War I. In the following years, on many occasions he had to change his line of research due to Nazi persecution. Lewy became a naturalized American, changed his name to Frederic Henry Lewey, and served in the US Army as a lieutenant colonel. Lewy died in 1950 and never used the famous eponym in his papers.

## Resumo

Em 1912, Friedrich Lewy descreveu os corpos de inclusão presentes na doença de Parkinson e na demência com corpos de Lewy. Ao longo sua vida, Lewy lutou em duas guerras, em lados opostos. Ele nasceu em Berlin em uma família de origem judaica e serviu no Exército alemão na Primeira Guerra Mundial. Nos anos seguintes, passou muitas vezes teve de mudar sua linha de pesquisa devido à perseguição Nazista. Lewy naturalizou-se americano, mudou seu nome para Frederic Henry Lewey, e serviu no Exército americano como tenente-coronel. Lewy morreu em 1950 e nunca usou o famoso epônimo em seus artigos.

## INTRODUCTION

**Figure 1:**
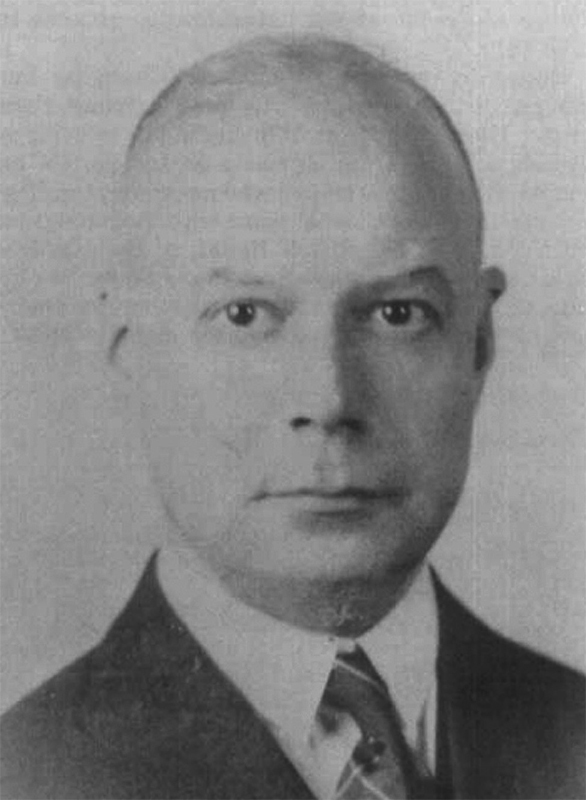
Dr. Lewy in 1934, following his immigration to the United States. Image extracted from Sweeney et al. 8

The twentieth century was a period of great development in Neurology. Friedrich Heinrich Jakob Lewy ( Figure 1 ) was an important neurologist in this period, famous because he was the first person to describe the inclusion bodies that bear his name (Lewy bodies) and that can appear in other types of dementia. 1 Moreover, the twentieth century was marked by two catastrophic wars, and these conflicts directly affected the scientists of the time, especially those of Jewish origin who lived in territories occupied by the Nazi regime.

## FREDERIC LEWY

The son of a Jewish physician in Berlin, Lewy was born on January 28, 1885. He started to conduct scientific research with Hermann Oppenheim, a pioneer of modern German neurology. 2 In 1910, he completed his medical course. In 1912, at the age o 25, he published the paper “Zur Pathologischen Anatomie der Paralysis agitans”, which made his name famous; in the paper, he described peculiar inclusions in neurons of the brain in paralysis agitans, later known as Parkinson disease. 3 4 While studying the substantia nigra in Parkinson disease, Gonzalo Lafora and Konstantin Tretiakoff named these inclusions Lewy bodies, creating the famous eponym. 1 From 1912 to 1914, Lewy was the director of the Neuropsychiatric Laboratory of Breslau University, together with Alois Alzheimer. 5

World War I forced Lewy out of the laboratory and onto the battlefield. He served the German Army as a military medical officer in France, Russia, and Turkey, and returned to Berlin at the end of the war. 2 Lewy became a Professor of Neurology in 1923 at Charité Hospital in Berlin, and was appointed director in 1932. One year later, Adolf Hitler was elected German Chancellor, and the Nazi regime dismissed Lewy in July 1933 because of his Jewish origin. 5

Lewy went to London in the summer of 1933 to escape antisemitism, and in the next year he emigrated to the United States with his wife Flora and his mother. He started working in the Rockefeller Foundation, where he was assigned to the hospital of the University of Pennsylvania, where he stayed until end of his career as Professor of Neuroanatomy and Associated Professor in Neuropathology. 5 6

European tensions were growing, and a new world war was imminent. In 1939, Lewy decided to anglicize his first names to suppress his German origins, changing Friedrich Heinrich to Frederic Henry. In 1940, he concluded his naturalization process and altered his surname from Lewy (/li.vi/) to Lewey (/lu.i/). In December 1941, after the Pearl Harbor attack, the United States entered World War II. The famous neurologist, now an American citizen, volunteered in the US Army Medical Corps as a lieutenant colonel and was again involved in a world war, but on the opposite side, fighting against the Nazi atrocities. He served as chief of the Neurology Section at Cushing General Hospital, a military hospital. 2 6

After the war, Lewy left the US Army and, in 1947, he became Professor of Neuropathology at the University of Pennsylvania, where he stayed until his sudden death on October 5th, 1950, at the age of 65. 2 6 Surprisingly, in his papers on paralysis agitans (1912-1924), Lewy focused more on cell loss in the striatum than on inclusion corpuscles. 7 Neither he nor the other German researchers during his lifetime ever used the eponym Lewy bodies. 7 The two World Wars and the difficulties imposed by the persecution of Jews made him change his line of research and caused many turbulent changes of workplace. It was only in the 1960s that diffuse cortical inclusion bodies, indicative of a condition different from Alzheimer disease, were identified. This condition was subsequently named Lewy body dementia. 2 3 More than half a century after he was buried in Pennsylvania, 8 Lewy bodies remain an important part of neurodegenerative diseases.
